# The Development of a Simulated Umbilical Line Insertion Model and Curriculum in the Neonatal Intensive Care Unit

**DOI:** 10.7759/cureus.13418

**Published:** 2021-02-18

**Authors:** Sunayna Gupta, Avery Longmore, Madeline Drake, Ra Han, Michael Sgro, Kathleen Hollamby, Douglas M Campbell

**Affiliations:** 1 Pediatrics, St. Michael’s Hospital, Unity Health Toronto, Toronto, CAN; 2 Pediatrics, University of Toronto, Toronto, CAN

**Keywords:** competency-based assessment, umbilical venous catheter, procedural skills, neonatology, simulation, residency training

## Abstract

Background

Insertion of an umbilical venous catheter (UVC) is a required skill for pediatric residents to learn and perform effectively. However, there is known variability in the ability of residents to perform this essential neonatal skill.

Objective

The objective of our study was to create a competency-based curriculum for umbilical vein catheter insertion using a human umbilical tissue simulated model, and to assess the feasibility of the curriculum on resident learners during their neonatology rotations.

Methods

We evaluated the curriculum by assessment of resident learning, reactions, and behaviours. Performance was assessed using the Ottawa Surgical Competency Operating Room Evaluation (O-SCORE).

Results

A total of 14 residents were included for analysis. The majority were ‘senior’ residents (postgraduate year (PGY)-3 and PGY-4 n = 10; PGY-1 n =4), and they reported a wide range of previous experience with UVC insertion prior to this curriculum implementation. The residents’ reaction to the curriculum was overwhelmingly positive. All residents maintained or improved in their knowledge assessment. O-SCORE results showed improvement in UVC insertion before and after curriculum completion for both junior (2.5 +/- 0.71 to 4.5 +/- 0.41) and senior (3.55 +/- 0.42 to 4.95 +/- 0.15, p < 0.001) residents. The mean improvement in O-SCORE was greater for junior residents than senior residents.

Conclusion

The results of this study demonstrate the feasibility and emerging impact of a competency-based curriculum using simulation for procedural skills.

## Introduction

Placement of an umbilical venous catheter (UVC) is an important skill for the resuscitation of critically ill newborns, and one of the most common procedures performed in neonatal intensive care units (NICUs) [1]. The insertion of umbilical catheters is a required skill for pediatric residents as determined by the Royal College of Physicians and Surgeons of Canada [2]. There is great variability, however, in clinical exposure to procedures among pediatric residents, and general pediatricians [3]. Residency program directors have questioned if residents receive adequate training in procedural skills during residency [4,5]. A 2007 survey of pediatric residency program directors showed that some of the current educational methods for procedural teaching were not sufficient for graduating residents to gain competence in important procedural skills [5].

Teaching umbilical line placement with the use of real umbilical cords has been described [6-10]. However, to our knowledge, it has not been implemented with applied educational theory. It has been suggested that the use of real umbilical cords for training is preferred over simulated umbilical cords given the higher fidelity [8], and face validity [9] of real tissue models. Pediatric residents may also prefer learning with real umbilical cord tissue [8,10]. Umbilical line teaching for residents in the NICU is often variable or non-standardized in our centre. Residents are occasionally taught UVC insertion with real umbilical cord simulation, however, there is no accepted formal methodology. Less than 40% of pediatric residency programs reported using simulation for teaching umbilical vein catheterization [5].

Residency curriculums in Canada have transitioned to a “Competency by Design” training model [11]. With this model, there is greater focus on demonstrating competence and independence in performing clinical skills. Clinical teachers are required to observe a skill, provide feedback, and document observations. It is imperative that we have frameworks for teaching essential neonatal skills, such as UVC insertion, within the framework of this model.

Our objective was to create a realistic simulation model using human umbilical tissue and develop a feasible competency-based curriculum. Kirkpatrick’s framework is an evaluation model employed in various fields to assess the impact and effectiveness of training interventions [12,13]. It proposes that an evaluation consists of assessments on four different levels: reaction, learning, behavior, and results [14]. These stages emphasize a learner’s ability to involve themselves without bias in new experiences, reflect on their experiences, cultivate their own theories based on their reflections, and finally utilize their newly developed theories to make decisions [15]. We also wanted to incorporate a ‘just-in-time’ teaching model through integrating in-person instruction and simulation-based education with deliberate practice, which has been shown to be superior to traditional teaching methods for skill acquisition [16].

We hypothesized that with the application of deliberate practice framework, Kolb’s cycle of Experiential Learning [15,17], and the utilization of Kirkpatrick’s framework for learning evaluations [14], residents will improve their ability to perform this skill, and progress towards competency. Ultimately, we hope that these educational endeavors will improve patient care for neonates requiring vascular access and improve resident training at our institution.

This article was previously presented as an oral presentation at IPSSW2019 (International Pediatric Simulation Symposia and Workshop) on May 21, 2019.

## Materials and methods

Pediatric, Family Medicine, and Obstetrics and Gynecology residents complete a one-month rotation through the NICU at St. Michael’s Hospital site of Unity Health Toronto in the University of Toronto Pediatrics Residency Program. Residents were identified a priori at the beginning of each teaching block and recruited for participation in the study by the education coordinator from September 2018 to June 2019. Residents who consented to participation were exposed to the new curriculum during the first week of their rotation. If residents chose not to give consent, they were permitted to participate in the curriculum but would not have any data recorded. This curriculum included a structured introductory teaching session, and a simulation session to attempt umbilical catheter insertion coupled with principles of deliberate practice (individual feedback and mastery). The teaching session was comprised of a knowledge pre-test, a didactic teaching session, and an instructional video. The simulation was comprised of simulated umbilical catheter insertion using human umbilical cord tissue with direct supervisor feedback, and personal reflection. This was repeated until the resident was able to perform the procedure safely and independently as judged by the evaluator. Once a trainee reached this point, the resident was deemed to have achieved competency. A knowledge post-test was also administered (Appendix A).

The curriculum was evaluated on multiple levels using Kirkpatrick’s Model as a framework [12]. Resident reaction was evaluated using a post-curriculum survey. Resident learning was assessed using a pre and post survey of knowledge. Resident performance was assessed using the Ottawa Surgical Competency Operating Room Evaluation (O-SCORE), which evaluates a trainee’s ability to perform the procedure safely and independently as determined by the evaluator [18]. The O-SCORE was administered at the first and the final UVC insertion by the teacher evaluator. This allowed us to assess improvement in UVC insertion for residents participating in the curriculum. We calculated the median score for each learner as an individual score. We then calculated the average of these individual scores for junior and senior residents. Two items on the O-SCORE evaluation were not applicable in a simulated scenario and were not included in the analyses (see Appendix B for all forms, including removed items from the O-SCORE). The resident’s level of training was used as a surrogate marker for experience (i.e. postgraduate year (PGY)1 = junior resident, PGY3/4 = senior resident) during analysis.

Values of significance for test results before and after the curriculum, and O-SCORE results were calculated using a one-tailed paired t-test. A value of significance was not performed for data pertaining to junior residents due to small sample size.

Written consent from parents was obtained for the use of a section of umbilical cord from discarded placentas post-delivery. This project was approved by the Research Ethics Board at St. Michael’s Hospital in Toronto, Ontario, Canada.

## Results

Demographics

A total of 15 residents participated in the study. All residents approached gave consent to participate. One resident was lost to follow-up with no data to report, so the total for analysis was n=14. There were 10 senior residents (8 PGY3s and 2 PGY4s), and four junior residents (PGY1s). All 10 senior residents were Pediatric residents. Among the junior residents, there was one Obstetrics and Gynecology resident, two Pediatric residents, and one resident who did not specify his or her specialty. We did not have complete data for all 14 residents, and therefore analyzed the data available, with numbers outlined in each section below. Before curriculum implementation, 3/14 participants (21.4%) had no prior experience with UVC insertion (all three were PGY-1 residents), and there was a wide range in terms of the number of previous insertions (range=0-30). During their rotations at St. Michael’s Hospital, 6/14 (42.9%) participants reported no opportunities for UVC insertion.

Resident reaction

The program was well received. When asked to rate their overall simulation experience, the median score for all residents (PGY-1 and PGY-3/4) was 5/5 (n = 14). The median score for the effectiveness of the simulation for improving UVC insertion skills was also 5/5 for all residents (n=14). Comments provided by the residents include:

“Simulated UVC insertion very useful to help consolidate limited experiences spread out over rotations. Although I felt I had a good grasp of the basics, the simulations helped to improve my knowledge of the details and reinforce my skill set.” (PGY-3)

“This was one of the best practice sessions. It is more realistic than using plastic models. The educational videos were well made, clear, and the in-person instruction was extremely helpful” (PGY-3)

“Very useful training, I feel much more confident doing a UVC when I will have to in the future. Awesome teaching." (PGY-1)

Resident learning

Eleven residents had test scores before and after the curriculum (8 PGY-3/4 residents, 3 PGY-1 residents). There was an improvement in mean resident scores on the knowledge test among PGY-1 and PGY-3/4 residents after curriculum implementation. For PGY-1 residents, the mean score on the knowledge test was 3/5 pre-curriculum, and 5/5 post-curriculum. For PGY-3 and PGY-4 residents, the mean score pre-curriculum was 3.6/5, and post-curriculum was 4.5/5 (p=0.055).

Resident performance

PGY-1 residents required a median of three simulations to achieve competency, while PGY-3/4 residents required a median of two simulations to achieve competency.

**Figure 1:**
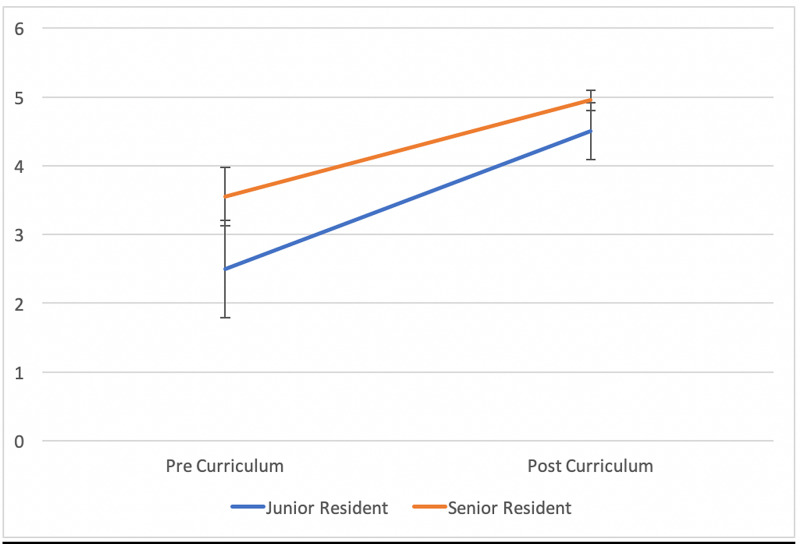
Comparison of Teacher Evaluated Modified O-SCORES Pre and Post-Curriculum Implementation The orange line represents the change in mean senior resident modified O-SCORES as assessed by the evaluators, and the blue line represents the change in mean junior resident modified O-SCORES assigned by the evaluators. Error bars represent standard deviation for each mean modified O-SCORE. O-SCORE: Ottawa Surgical Competency Operating Room Evaluation.

Thirteen residents had teacher evaluator O-SCORE results at the beginning and at the end of the curriculum (3 PGY-1, 10 PGY 3/4). The mean modified O-SCORE pre-curriculum was 2.5 (+/- 0.71) and post-curriculum was 4.5 (+/- 0.41) for PGY-1 residents. For PGY-3/4 residents, the mean modified teacher evaluated O-SCORE pre curriculum was 3.55 (+/- 0.42) and post curriculum was 4.95 (+/- 0.15) (p < 0.001) (Figure 1).

## Discussion

The results of this study demonstrate that implementation of a competency-based curriculum for residents using simulation is feasible and impactful to trainees. Opportunities to practice skills and develop procedural competencies are varied amongst resident trainees. This was demonstrated in our study by the wide range reported for previous experience with umbilical catheter insertion.

Knowledge and performance of UVC insertion improved after the curriculum for both junior and senior residents. The improvement in teacher evaluated O-SCORE results for PGY-3/4 residents before and after curriculum completion reached a significant value. This demonstrates that the residents improved in their confidence and ability to complete a UVC insertion while participating in the curriculum. During post-curriculum reflections, many of the residents qualitatively reported that the experience was a helpful practice to solidify skills and improve their confidence in performing UVC insertions for patients in the future. Both junior and senior residents expressed that the curriculum was beneficial in acquiring skills in UVC insertion or building on their previous experiences.

Pediatrics residents have reported that they feel inadequately prepared to perform required procedures and respond in situations like resuscitations by the end of their residencies [19,20]. Similarly, pediatric program directors in both Canada and the United States have reported that many residents may not develop the competency to perform several important procedures by the end of residency [4,5]. This highlights the importance of a shift toward competency-based medical education to define the milestones learners will need to achieve on their road to competency [21].

Use of human umbilical cord tissue to teach UVC insertion has been described in the literature [6-10]. However, to our knowledge, this is one of the first studies to embed educational theory in the development and evaluation of a curriculum using real umbilical cords for UVC insertion teaching.

With respect to Kirkpatrick’s Four Levels of analysis [12,14], our study showed that the overall reaction to the simulated curriculum was positive. Resident learning was evaluated with a multiple-choice UVC insertion knowledge test, and our results demonstrate evidence of knowledge acquirement and improvement through participation in our curriculum, although this improvement did not reach significance.

Simulated assessments of resident behavior and application of their knowledge to the clinical environment were evaluated using the O-SCORE. Our results demonstrate that the curriculum had a positive impact on residents’ ability to apply their knowledge during simulation, as both junior and senior residents had improvements in their post-curriculum O-SCOREs. Some were able to further apply the knowledge they acquired clinically, but most did not have the opportunity in our study due to time limitations and scope of follow-up.

There are limitations to our study. First, our sample size was small, and therefore we were not always able to accurately estimate differences in resident performances before and after curriculum implementation. Our sample was also specific to the residency program at the University of Toronto and may not be generalizable to other programs. The O-SCORE has not been previously validated for UVC insertion, and therefore, use in larger studies would be of value in improving face validity.

Future studies in this area are needed to determine which parts of the curriculum were most useful. Additionally, longitudinal evaluations over the entire residency would be beneficial to assess which areas contribute to sustainability of the skill.

## Conclusions

In conclusion, UVC insertion is a crucial skill for physicians caring for the neonate, and it is important that residents demonstrate safe, effective, and appropriate performance of this skill during training. This study demonstrated the feasibility of a simulated curriculum. As well, residents demonstrated an improvement in their performance of UVC insertion through completing the curriculum. Next steps should include further widespread implementation of a simulated competency-based curriculum and analysis of impact on clinical care.

## Figures and Tables

**Figure 2:**
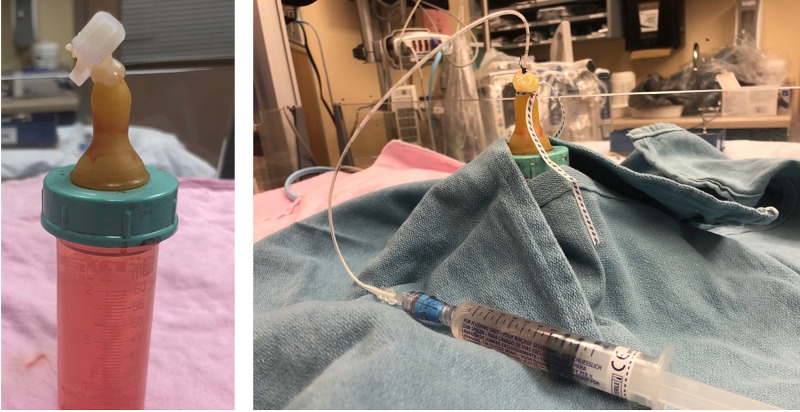
Umbilical Venous Catheter (UVC) Simulation Model Before and After UVC Insertion UVC simulation model before and after UVC insertion. Pictured on the left is a simulated umbilical cord using human umbilical tissue. Pictured on the right is a completed simulated UVC insertion.

**Figure 3:**
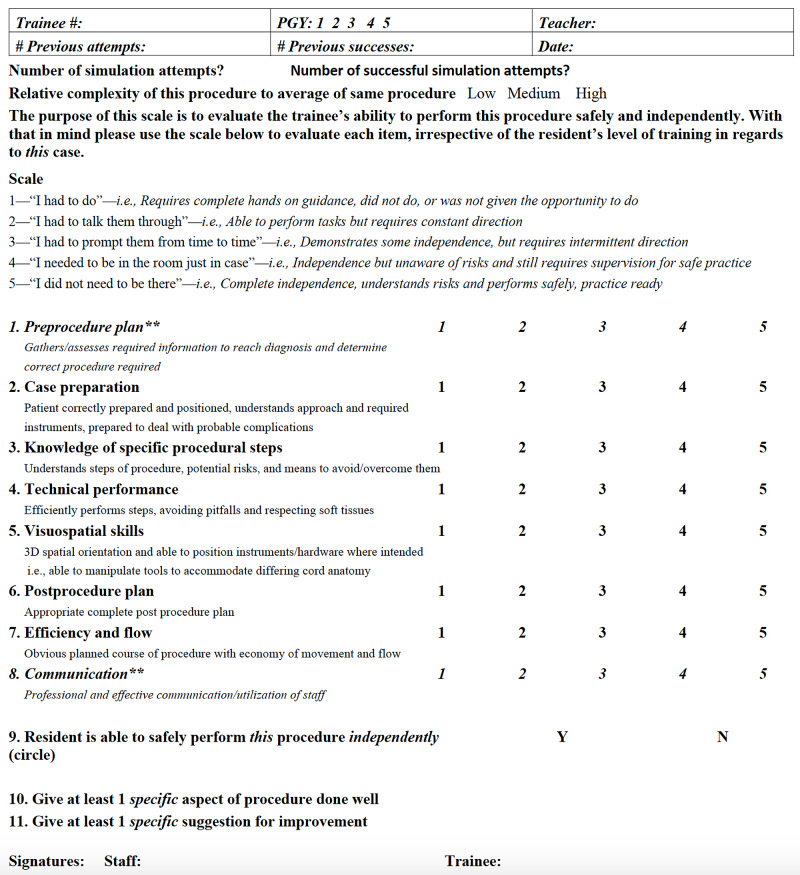
Modified Ottawa Surgical Competency Operating Room Evaluation (O-SCORE) for UVC Insertion Teacher Form **Removed from evaluation because determined to be not applicable to this simulation exercise Adapted from [18]

**Figure 4:**
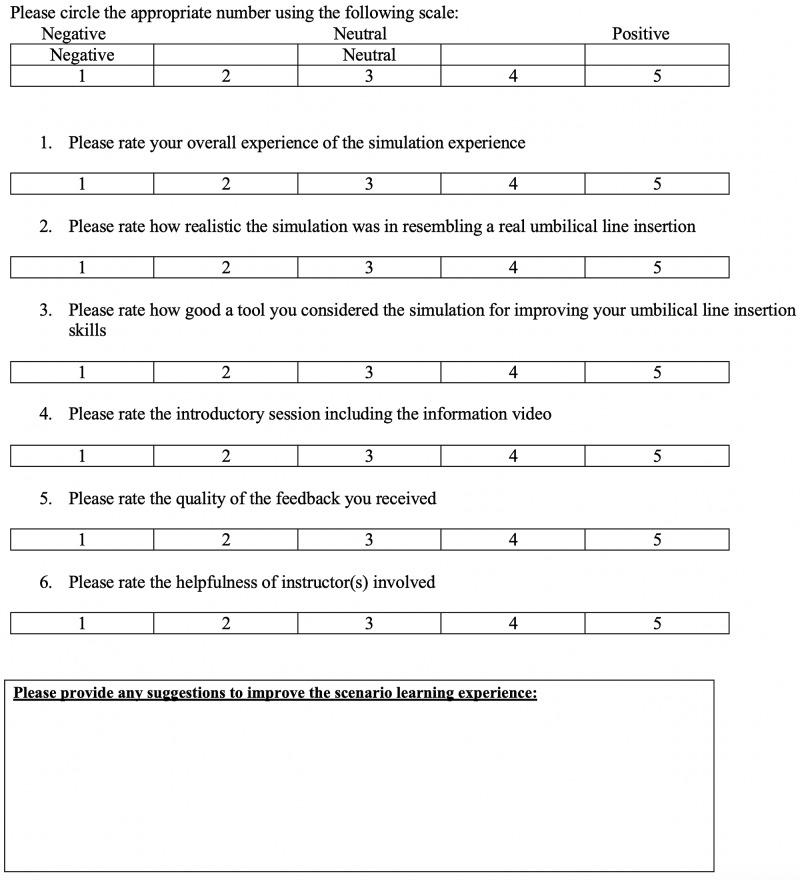
Resident Reaction/Perception of Simulation Exercise Adapted from [22]

## Appendices

Appendix A

Procedure to Complete UVC Curriculum

1. Obtain consent from learner to participate in the study

2. Have learner complete knowledge assessment test

3. Complete didactic curriculum. This includes a video demonstration and verbally reviewing indications, contraindications, and steps of UVC insertion

4. Obtain consent from the parent to use umbilical tissue

5. Prepare equipment and develop the simulation field, with the umbilical cord in a bottle (Figure 2)

6. Resident to complete UVC insertion in a simulated environment (Figure 2)

7. Evaluator to complete O-SCORE assessment

8. Repeat steps 4-7 through resident’s one-month rotation, until resident achieves competency in skill

9. After final UVC insertion, evaluator to complete O-SCORE assessment again

10. Resident to complete resident reaction form, and repeat knowledge assessment test

Appendix B

Umbilical Venous Line Knowledge Assessment Test Pre/Post

1. You attend the delivery of a 31+2 week baby boy. It is an uncomplicated pregnancy and baby is born by SVD with APGARS 9 and 9 at 1 and 5 minutes respectively. Baby develops increased work of breathing and is started on CPAP. Baby is now stable on CPAP room air, PEEP 5. Birth weight is 1600g. Which of the following is the best next step?

a. Insert an umbilical venous catheter to start TPN

b. Insert an umbilical venous catheter to prepare for intubation

c. Insert an umbilical artery catheter for frequent blood work

d. Insert a peripheral venous catheter for iv dextrose

2. Which of the following is the best indication for umbilical venous line insertion?

a. Oomphalocele

b. Necrotizing enterocolitis

c. Prematurity at 29 weeks gestation

d. Hypoglycemia requiring IV dextrose

3. Which of the following is a complication of umbilical venous line insertion?

a. Pneumonia

b. Cardiac arrhythmia

c. Hepatic hematoma

d. Pneumothorax

4. What is the correct depth for catheter placement in a 2.5 kg neonate? 

a. 7cm

b. 8cm

c. 9cm

d. 10cm

5. A chest and abdominal x-ray is performed to assess umbilical venous line placement. Which of the following best matches the correct placement of the line?

a. The tip of the catheter is just inside the border of the heart at T9

b. The tip of the catheter is just above the diaphragm at T10

c. The tip of the catheter is just below the diaphragm at L1

d. The tip of the catheter is just inside the liver

Modified Ottawa Surgical Competency Operating Room Evaluation (O-SCORE) for UVC Insertion Teacher Form (Figure 3)

Resident Reaction/Perception of Simulation Exercise (Figure 4)
